# Exploring Biocontrol Agents From Microbial Keystone Taxa Associated to Suppressive Soil: A New Attempt for a Biocontrol Strategy

**DOI:** 10.3389/fpls.2021.655673

**Published:** 2021-03-19

**Authors:** Yanfen Zheng, Xiaobin Han, Donglin Zhao, Keke Wei, Yuan Yuan, Yiqiang Li, Minghong Liu, Cheng-Sheng Zhang

**Affiliations:** ^1^Pest Integrated Management Key Laboratory of China Tobacco, Tobacco Research Institute of Chinese Academy of Agricultural Sciences, Qingdao, China; ^2^Biological Organic Fertilizer Engineering Technology Center of China Tobacco, Zunyi Branch of Guizhou Tobacco Company, Zunyi, China

**Keywords:** bacterial wilt disease, microbiome, Biocontrol Agents, network, biocontrol, suppressive soil

## Abstract

Recent studies have observed differing microbiomes between disease-suppressive and disease-conducive soils. However, it remains unclear whether the microbial keystone taxa in suppressive soil are critical for the suppression of diseases. Bacterial wilt is a common soil-borne disease caused by *Ralstonia solanacearum* that affects tobacco plants. In this study, two contrasting tobacco fields with bacterial wilt disease incidences of 0% (disease suppressive) and 100% (disease conducive) were observed. Through amplicon sequencing, as expected, a high abundance of *Ralstonia* was found in the disease-conducive soil, while large amounts of potential beneficial bacteria were found in the disease-suppressive soil. In the fungal community, an abundance of the *Fusarium* genus, which contains species that cause *Fusarium* wilt, showed a positive correlation (*p* < 0.001) with the abundance of *Ralstonia*. Network analysis revealed that the healthy plants had more complex bacterial networks than the diseased plants. A total of 9 and 13 bacterial keystone taxa were identified from the disease-suppressive soil and healthy root, respectively. Accumulated abundance of these bacterial keystones showed a negative correlation (*p* < 0.001) with the abundance of *Ralstonia*. To complement network analysis, culturable strains were isolated, and three species belonging to *Pseudomonas* showed high 16S rRNA gene similarity (98.4–100%) with keystone taxa. These strains displayed strong inhibition on pathogens and reduced the incidence of bacterial wilt disease in greenhouse condition. This study highlighted the importance of keystone species in the protection of crops against pathogen infection and proposed an approach to obtain beneficial bacteria through identifying keystone species, avoiding large-scale bacterial isolation and cultivation.

## Introduction

Bacterial wilt, a common soil-borne disease caused by *Ralstonia solanacearum*, occurs annually in most tobacco cultivation regions and causes huge economic losses (Jiang et al., 2017a). It has been reported that plants can adjust their microbiome and specifically recruit disease-resistant and growth-promoting microbes or microbial consortia when they encounter a disease (Berendsen et al., 2018). The soil with low disease incidence even in the coexistence of susceptible plants and pathogens was defined as disease-suppressive soil. The mechanisms behind soil-mediated suppression of diseases, such as bacterial wilt disease in tobacco (Qi et al., 2019; Zhang et al., 2020), *Fusarium* wilt in vanilla (Xiong et al., 2017), *Rhizoctonia solani* in sugar beet (Mendes et al., 2011), and black root rot in tobacco (Kyselkova et al., 2009), have been extensively investigated. The soil microbiome has been deciphered in detail, and it has been found that the soil compositions between suppressive and conducive soils are distinct, although they share similar geographical and climatic conditions, as well as the same agronomic management.

To prevent and control bacterial wilt disease, hundreds of beneficial microbes that exhibit suppressive activity against *R. solanacearum* have been isolated (Jiang et al., 2017a), e.g., *Pseudomonas* spp. (Raaijmakers and Weller, 1998; Mendes et al., 2011; Hu et al., 2016), *Bacillus* spp. (Wei et al., 2011; Guo et al., 2020), *Burkholderia* spp. (Nion and Toyota, 2008), and *Streptomyces* spp. (Boukaew et al., 2011). They control bacterial wilt disease by producing antibacterial substance and inducing host systemic resistance. Moreover, it has been indicated that multiple beneficial microbial consortia or synthetic community (SynCom) has been shown to be more efficient than the single isolates in controlling bacterial wilt (Hu et al., 2016; Jiang et al., 2017a), suggesting that interactions and synergistic effects among microbes are critical.

Network analysis is a particularly useful tool to understand microbe-microbe associations and to visualize co-occurrence patterns among communities (Barberan et al., 2012; de Vries et al., 2018; Banerjee et al., 2019). Previous studies have shown distinct bacterial networks between healthy and diseased plants (Xiong et al., 2017; Wei et al., 2019). The bacterial network of healthy soil was more complex and stable than bacterial wilt-susceptible soil (Qi et al., 2019). Network scores can be used to identify keystone taxa, i.e., species highly associated with a microbial community (Berry and Widder, 2014; Banerjee et al., 2018), which have a significant influence on the community (Agler et al., 2016; Banerjee et al., 2016; Hu et al., 2020). Extensive keystone taxa have been identified in various terrestrial ecosystems, such as forest (Ding et al., 2015), agricultural (Jiang et al., 2017b; Wang et al., 2017a), and grassland soils (Lupatini et al., 2014). These keystone taxa can be indicators of plant or soil microbiome shifts linked to abiotic factors (Zhou et al., 2011; Liang et al., 2016) and host genotypes (Jiang et al., 2017b). Moreover, Agler et al. have suggested that microbial keystone taxa are promising targets in control of plant-pathogen relationships and in construction of beneficial host-associated microbial communities (Agler et al., 2016). A previous study showed that keystone taxa contributed to the divergences in the microbiome between healthy and diseased tomato status and demonstrated that the *Massilia*, *Dyadobacter*, *Terrabacter*, *Arachidicoccus*, and *Dyella* genera were potential keystone taxa in healthy plants (Wei et al., 2019). Qi et al. found more key microorganisms (e.g., *Bacillus* and *Actinobacteria*) in healthy soil than in bacterial wilt-susceptible soil through network analysis. However, it remains unclear if these keystone microbial species have the ability to suppress bacterial wilt. Additionally, previous studies mainly focused on bacterial community and network structure, but the impact on fungal composition and network made by bacterial wilt disease are little known.

In this study, we observed two contrasting fields. Bacterial wilt disease incidence was 0% in suppressive soil but 100% in conducive soil, although they were geographically proximate and shared the same management. Here, we aimed to answer the following: (i) How does the bacterial wilt disease change the microbe-microbe interaction and network structure? (ii) What is the keystone taxa in suppressive soil? (iii) What is the relationship between keystone taxa and bacterial wilt disease pathogen? (iv) Can these keystone taxa reduce the incidence of bacterial wilt disease? To achieve this, we sampled tobacco plants from these contrasting fields. Bacterial and fungal communities were determined by 16S ribosomal RNA (rRNA) gene and internal transcribed spacer (ITS) amplicon sequencing, respectively. Network analyses were used to uncover microbe-microbe interactions and identify keystone taxa from suppressive fields. Culturable keystone species were isolated, and their role in protecting against disease was evaluated using a greenhouse experiment.

## Materials and Methods

### Sampling

We collected 40 samples from four model experiment stations in Zunyi city (Guizhou province, China), all of which harbored two contrasting soils, bacterial wilt disease-suppressive and disease-conducive soils. Bacterial wilt disease rarely occurred in disease-suppressive soil, although tobacco had been continuously planted for ~20 years. In disease-conducive soil, high disease incidences were observed every year. Rhizosphere soil and root samples were collected from each field on August 10, 2018 (70 days after transplanting). When we collected samples, none of the plants showed wilt symptoms in the suppressive soil, while all plants suffered from different levels of wilt disease in the conducive soil. Thus, healthy plants were collected from the suppressive soil, and plants with slight disease symptoms were collected from the conducive soil. From each field, two or three random tobacco plants were chosen. The roots were shaken to remove loosely adhering soil, put into sterile plastic bags, and transported to the laboratory on ice. Finally, the four groups in the study were rhizosphere soil (SS, *n* = 10) and healthy tobacco roots (HR, *n* = 10) in suppressive fields and rhizosphere soil (CS, *n* = 10) and infected tobacco roots (IR, *n* = 10) in conducive fields.

### Sample Processing and DNA Extraction

Soil attached firmly to the root was collected with a brush and considered to be the rhizosphere soil. The root was shaken vigorously with phosphate-buffered saline (PBS) solution in order to clean all the soil from the root surface. They were then soaked in alcohol (75%) for 2 min and sodium hypochlorite (5%) for 5 min. The final rinse with sterile water was performed three times for 1 min. We refer to the community on surface-sterilized roots as the root microbiome. All samples were stored at −80°C until DNA extraction. Rhizosphere soil (0.5 g) and root samples were used to perform DNA extractions with the DNeasy® PowerSoil Kit (Qiagen) according to the manufacturer’s instructions. DNA concentration and purity were monitored using a nanodrop and 1% agarose gels, respectively.

### PCR Amplification and 16S rRNA Gene Amplicon Sequencing

For the community analysis, the V5–V7 regions of the 16S rRNA gene and the ITS region 1 of the nuclear ribosomal coding cistron were amplified using primers 799F and 1193R and ITS1F and ITS2R (Supplementary Table S1). The PCR reactions were performed in 30 μl reactions with 15 μl of the Phusion® High-Fidelity PCR Master Mix (2×, New England Biolabs), 0.2 μM of primers, and 10 ng of the template DNA. Thermal cycling for the 16S rRNA gene consisted of an initial denaturation at 98°C for 1 min, followed by 30 cycles of 98°C for 10 s, 55°C for 30 s, and 72°C for 30 s, and a final extension step at 72°C for 5 min. Thermal cycling for ITS region 1 consisted of 95°C for 5 min, followed by 34 cycles of 94°C for 1 min, 57°C for 45 s, 72°C for 1 min, and a final extension step at 72°C for 10 min. PCR products were detected using 2% agarose gel electrophoresis. Then, the PCR product mixture in equi-density ratios was purified with the GeneJETTM Gel Extraction Kit (Thermo Scientific). Libraries were constructed using the Ion Plus Fragment Library Kit 48 rxns (Thermo Scientific). The qualified libraries were sequenced on an Ion S5TM platform (Thermo Fisher Scientific Inc., Waltham, MA, United States) at Novogene Bioinformatics Technology Co., Ltd. (Beijing, China).

### Quantification of Bacteria and *R. solanacearum*

Bacterial abundance was determined using the Eub338F/Eub518R primers (Supplementary Table S1). The specific primer Rsol_*fliC* (Supplementary Table S1) that targets the *fliC* gene encoding the flagellum subunit was used to quantify *R. solanacearum* densities. The quantitative PCR (qPCR) analyses were performed using an Applied Biosystems 7,500 Real-Time PCR System (Applied Biosystems, United States). Standard curves were generated using 10-fold serial dilutions of a plasmid containing the *16S rRNA* gene from *Arthrobacter pokkalii* and a fragment copy of *R. solanacearum fliC*. qPCR amplifications for standard and DNA samples included a 20-μl mixture containing 2 μl of templates, 10 μl of the SYBR Green premix Ex Taq (2×), 0.4 μl of ROX Reference Dye II, 0.4 μl of each primer, and distilled water. The PCR thermal cycling conditions for bacteria and *R. solanacearum* were conducted according to previous procedures (Muyzer et al., 1993; Schonfeld et al., 2003; Fierer et al., 2005) with three technical replicates.

### Amplicon Sequence Processing and Statistical Analysis

Raw reads were quality filtered using Cutadapt v1.9.1 (Martin, 2011) to remove low-quality reads and obtain high-quality clean reads. Chimera sequences were detected using the UCHIME algorithm (Edgar et al., 2011) and then removed to obtain the final clean reads. Operational taxonomic unit (OTU) assignment was performed by Uparse (v7.0.1001; Edgar, 2013) at a similarity of 97%. For each OTU representative sequence, the Silva (release 132, https://www.arb-silva.de) and Unite database (version 7.2, https://unite.ut.ee) were used to annotate bacterial and fungal taxonomic information, respectively. To equalize sequencing depth, each sample was rarefied to the lowest sequence number across all samples (41,326 reads for bacteria and 63,022 reads for fungi) for downstream analyses. Alpha and beta diversities were calculated with Qiime (version 1.7.0; Caporaso et al., 2010) and visualized using R software.

Differences between the microbial communities of the suppressive and conducive field samples (including soil and root) were calculated using analysis of similarities (ANOSIM) in PRIMER 6 (Clarke and Gorley, 2006). The microbiome composition in the different samples was ordinated by principal coordinates analysis (PCoA) by unweighted UniFrac distance with WGCNA package, stat packages, and the ggplot2 package in R software. In this study, the top 100 abundant bacterial genera were used for co-occurrence network construction. The Spearman correlation matrix was calculated [absolute correlation coefficient values >0.6; the false discovery rate (FDR) adjusted *p* < 0.05] using the psych package in the R environment (Revelle, 2017). The network was analyzed and visualized using Gephi (version 0.9.2; Bastian et al., 2009). The taxa harboring the highest degree and closeness centrality and the lowest betweenness centrality values were considered as the keystone taxa (Berry and Widder, 2014). Based on this, genera with a degree >40, a closeness centrality >0.61, and a betweenness centrality <0.18 were considered as the keystone taxa for the soil and root in suppressive fields. We further used the online NetShift tool,^1^ which could identify potential driver taxa that maintain the healthy microbiome against diseased samples. Statistical analyses were performed using *t* tests in GraphPad Prism 7. Linear regression analysis was performed in Microsoft Excel to determine the association between the absolute abundance (qPCR) and relative abundance (amplicon sequencing) of *R. solanacearum* as well as the abundances of *Ralstonia* and *Fusarium*.

### Bacterial Isolation and Identification

Surface sterile root samples from the suppressive soil were ground. The homogenate was serially diluted to 10^−2^, and 100 μl of each dilution was spread on nutrient agar (NA) for bacterial isolation. Media were prepared according to methods described by Dhingra and Sinclair (1985). All plates were incubated at 28°C for 2–4 days. Morphologically different bacterial isolates were picked from plates containing approximately 30–300 colonies. All the isolates were purified three times and stored at −80°C with 15% (v/v) glycerol. The bacterial genomic DNA was extracted using an EasyPure Bacteria Genomic DNA Kit according to the manufacturer’s instructions. Bacterial universal primers 27F/1492R (Supplementary Table S1) were used to amplify the full-length 16S rRNA genes. PCR products were tested by 1.0% agarose gel electrophoresis and sequenced by TsingKe Biological Technology Co., Ltd. (Beijing, China). Identification and sequence similarity were achieved using the National Center for Biotechnology Information (NCBI; https://www.ncbi.nlm.nih.gov/). Sequences of these isolates and OTUs from high-throughput sequencing were aligned using the CLUSTAL_X program (Thompson et al., 1997), and a neighbor-joining phylogenetic tree was constructed using MEGA 7.0 with a bootstrap value of 1,000 replicates (Kumar et al., 2016).

### Inhibition and Greenhouse Experiment

Three isolates (*Pseudomonas lurida* FGD5-2, *Pseudomonas koreensis* HCH2-3, and *Pseudomonas rhodesiae* MTD4-1) that showed high 16S rRNA gene similarity with keystone taxa were selected. Their inhibitory effect on *R. solanacearum* were determined by the Oxford cup method. Briefly, 100 μl of *R. solanacearum* culture was spread on NA plates. Then, sterilized Oxford cups were placed on the plates, and 200 μl culture of each isolate was put into these cups in triplicate. All plates were incubated at 28°C for 24 h. The diameter of the inhibition zone was recorded. For the greenhouse experiment, tobacco seeds were sown into a seedling tray. At the three-leaf stage, tobacco plants were transplanted to a plastic pot containing 300 g of soil collected from a farmland in Qingdao city, Shandong province, China. Bacteria were cultivated using nutrient broth medium for 24 h and centrifuged at 4°C, 4000 rpm to concentrate bacterial cells and then resuspended them in the sterile water. After 5 days of transplantation, bacterial cells were inoculated by the use of root drenching methods with a final density of ~10^7^ cells per gram of soil three times at intervals of 5 days (Wei et al., 2013). Microbial consortia, a mixture of all isolates with a 1:1 ratio, were also inoculated. The control group was treated with equal amounts of sterile water. After 5 days postinoculation of beneficial stains, the pathogen of *R. solanacearum* Rs10 (prepared as the same methods of keystone species) was inoculated, resulting in ~10^7^ cells/g soil. Each treatment was replicated three times, and each replicate contained 12 plants. Plants were arranged in a climate chamber at 30°C with a relative humidity of 70% and watered regularly with sterile water. The disease index was assessed 15 days after pathogen inoculation.

## Results

### The Abundances of Bacteria and *R. solanacearum*

Based on the quantitative PCR results, the bacterial 16S rRNA gene abundance ranged from 6.15 × 10^8^ to 1.51 × 10^9^ copies/g. No significant differences in bacterial abundance were observed among the different samples (Supplementary Figure S1). As expected, the densities of *R. solanacearum* increased significantly (*p* < 0.01) with 5.12 × 10^6^ and 9.13 × 10^7^ copies of *fliC* gene per gram in conducive soil and infected roots, respectively. These values in suppressive soil and healthy roots were 2.39 × 10^4^ and 6.86 × 10^4^ copies/g, respectively (Supplementary Figure S1). Additionally, the fold changes in *R. solanacearum* densities between the suppressive and conducive fields were larger in the roots (~1,330-fold) than in the soil (~214-fold), suggesting that *R. solanacearum* infection might exert more of an impact on the root compartment.

### Diversity of Microbial Communities

**Figure 1:**
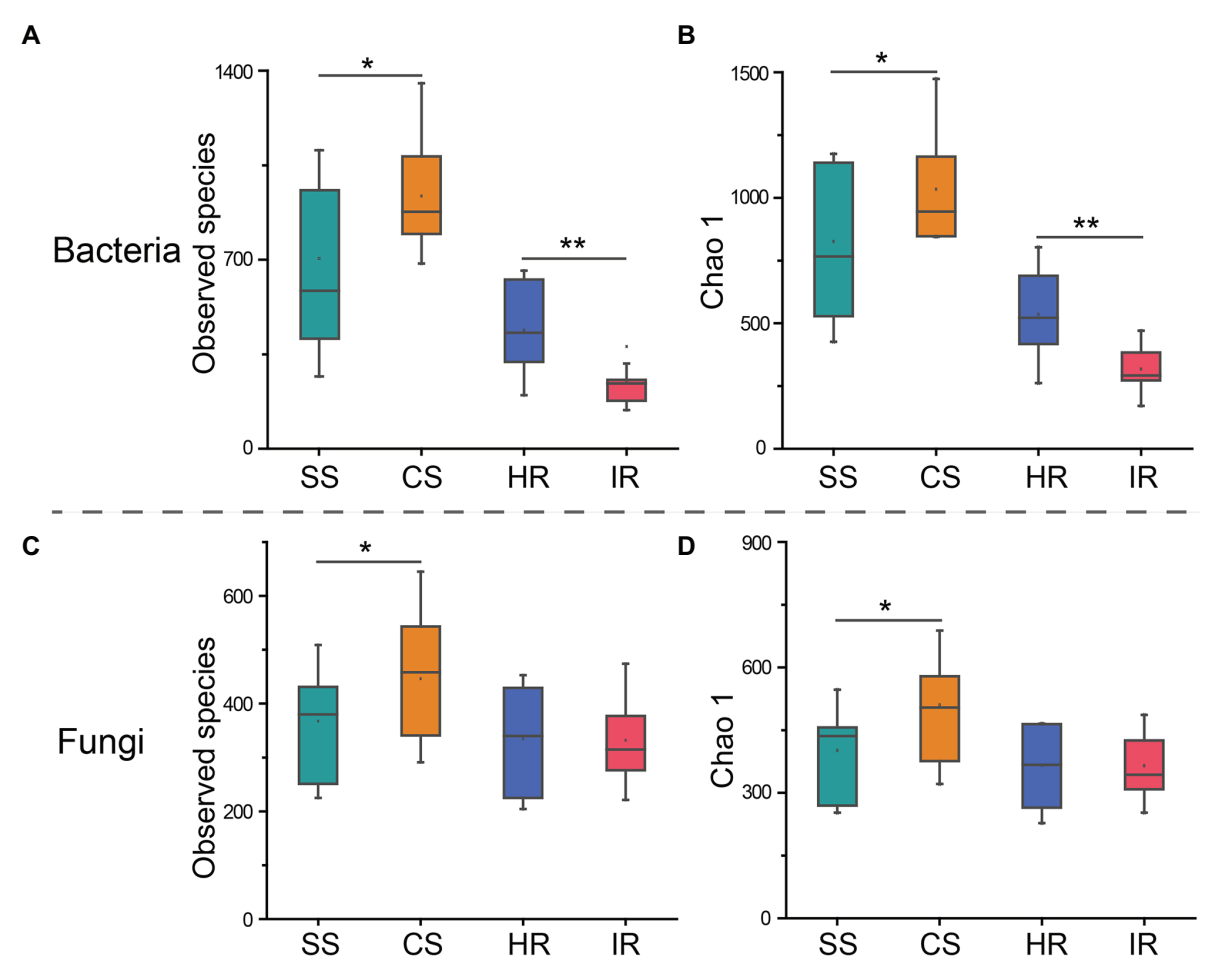
Alpha diversity indices of bacteria and fungi in different samples. **(A)** Bacterial operational taxonomic unit (OTU) numbers, **(B)** bacterial Chao 1, **(C)** fungal OTU numbers, and **(D)** fungal Chao 1. SS, suppressive rhizosphere soil; CS, conducive rhizosphere soil; HR, root of healthy tobacco; IR. root of infected tobacco. The asterisks (^*^) indicate significant differences determined by the Student’s *t* test. ^*^p < 0.05; ^**^p < 0.01.

A total of 3,798,025 and 3,846,278 clean reads were produced from 16S rRNA gene and ITS amplicon sequencing, respectively. Good’s coverage of all samples was >99.4%, and most rarefaction curves tended to reach the saturation plateau (Supplementary Figure S2), which suggested that the sequencing depth was sufficient to represent the microbial community in these environments. These sequences were grouped into 2,469 bacterial and 1,606 fungal OTUs. The Venn diagram showed that 591 bacterial OTUs were shared among the four groups, while 248, 286, 63, and 9 OTUs were unique to suppressive soil, conducive soil, healthy root, and infected root, respectively. The four groups shared 499 fungal OTUs, while suppressive soil, conducive soil, healthy root, and infected root harbored 106, 198, 93, and 104 specific OTUs, respectively (Supplementary Figure S3). According to the diversity indices, we observed that both bacterial and fungal species were significantly lower (*p* < 0.05) in the suppressive soil than in the conducive soil (Figures 1A,C). In contrast, for the root samples, the observed bacterial species in the healthy plants were significantly higher (*p* < 0.001) than those in the infected plants, whereas there was no significant difference in fungal species. These diversity patterns were confirmed by the Chao1 indices (Figures 1B,D).

**Figure 2:**
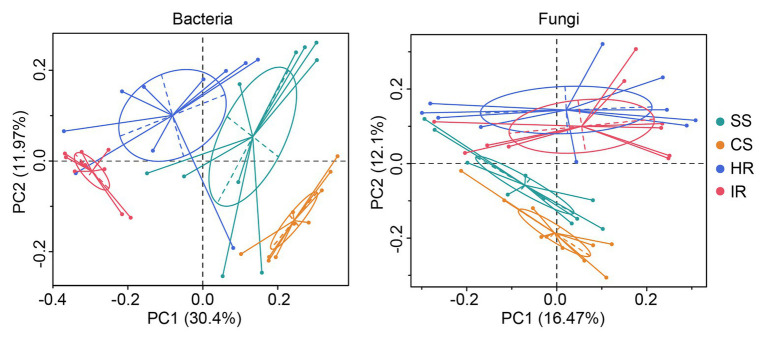
Principal coordinate analysis of the bacterial and fungal communities in rhizosphere soil and root based on unweighted UniFrac distance. SS, suppressive rhizosphere soil; CS, conducive rhizosphere soil; HR, root of healthy tobacco; IR, root of infected tobacco.

For the beta diversity, principal coordinate analysis (PCoA) showed that both the bacterial and fungal communities from the suppressive soil and healthy root were clearly separated from the conducive soil and infected root (Figure 2). This result was confirmed by the analysis of similarity (ANOSIM) of Bray-Curtis measures, which indicated that significant differences were observed between the suppressive and conducive soils (*R* = 0.530, *p* < 0.01 for bacteria; *R* = 0.339, *p* < 0.01 for fungi) and the healthy and infected roots (*R* = 0.656, *p* < 0.01 for bacteria; *R* = 0.553, *p* < 0.01 for fungi). Additionally, there were no significant differences between the soil and root of healthy plants (*R* = 0.0408, *p* = 0.203) as well as the soil and root of infected plants (*R* = 0.0644, *p* = 0.147) regarding the fungal community, suggesting that a healthy status rather than the sample type was the key factor affecting the fungal profile in this study.

### Specific Differences in Microbiome Between Suppressive and Conducive Fields

**Figure 3:**
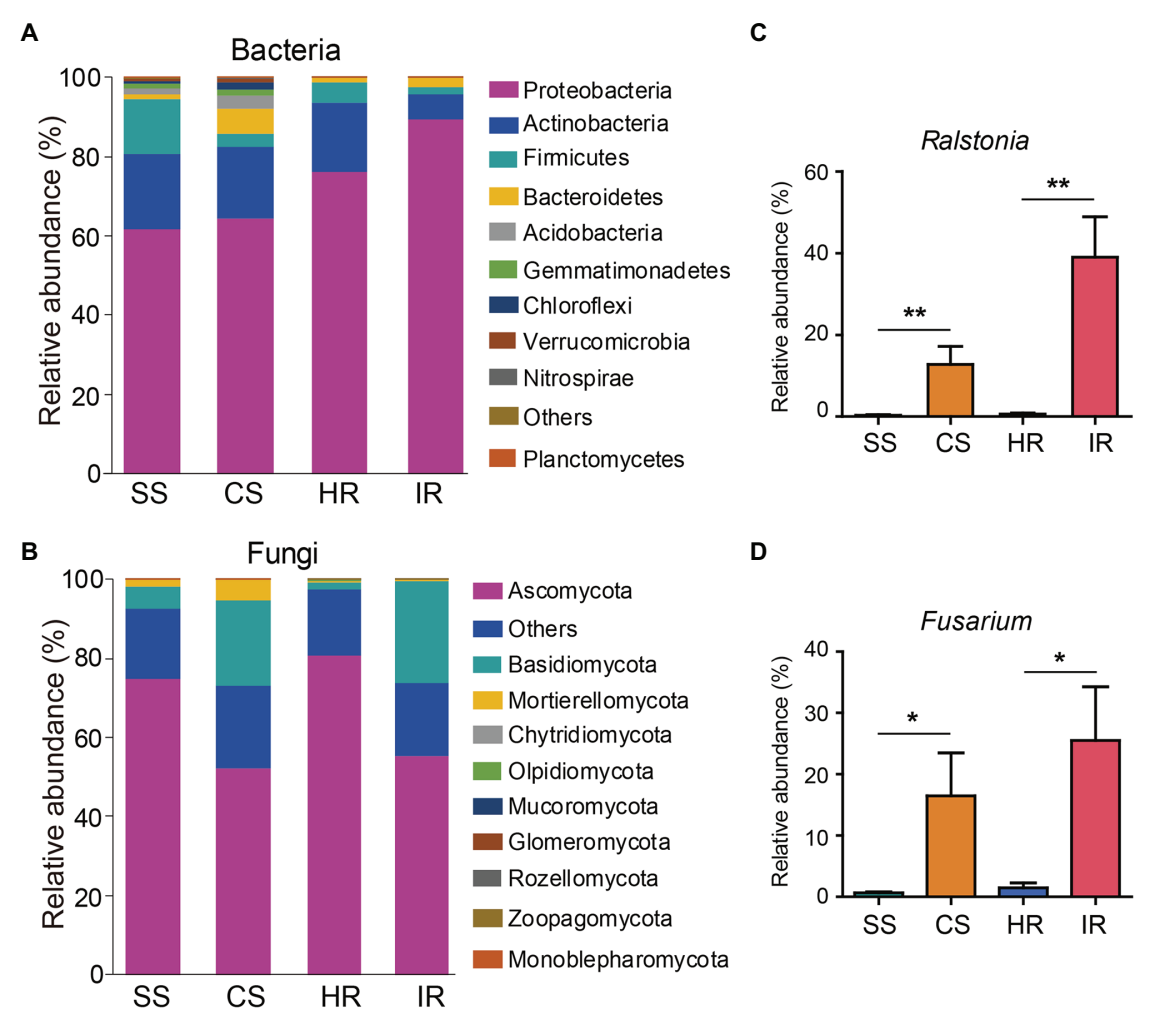
Changes in the rhizosphere soil and root microbiome composition in different samples. **(A)** Bacterial community at the phylum level. **(B)** Fungal community at the phylum level. The relative abundances of Ralstonia and Fusarium in different samples were shown in Panels (**C** and **D**), respectively. Bars represent the standard error of the mean, and asterisks (^*^) indicate significant differences determined by the Student’s *t* test. ns, no significant; ^*^p < 0.05; ^**^p < 0.01. SS, suppressive rhizosphere soil; CS, conducive rhizosphere soil; HR, root of healthy tobacco; IR, root of infected tobacco.

At the phylum level, all samples shared three dominant bacterial phyla, Proteobacteria, Actinobacteria, and Firmicutes, accounting for 61.5–89.3%, 6.4–19.0%, and 1.9–13.7% of the total bacterial communities, respectively. However, these three phyla varied in their relative abundance in the different samples. More Firmicutes and less Proteobacteria and Bacteroidetes were observed in the suppressive soil than in the conducive soil, and a similar trend was found in the root samples (Figure 3A). The most dominant phyla in the fungal community were Ascomycota (52.1–80.8%), Basidiomycota (1.8–25.9%), and Mortierellomycota (0.3–5.0%). Ascomycota were more abundant in the suppressive soil and healthy roots than in the conducive soil and infected roots, whereas Basidiomycota showed the opposite trend (Figure 3B).

To further investigate the taxa in order to discriminate between the microbiomes of the different samples, MetaStat analysis was performed based on the relative abundance of the microbial community at the genus level (Supplementary Figure S4). The relative abundance of the *Ralstonia* genus, which contains the pathogenic species of bacterial wilt disease, was much higher in the conducive soil (12.8%) and infected root (39.0%) than in the suppressive soil (0.3%) and healthy root (0.6%; Figure 3C). Although we could not attest that all OTUs assigned to *Ralstonia* in this study were pathogenic species, the relative abundance of *Ralstonia* from 16S rRNA gene amplicon sequencing correlated positively (*R*^2^ = 0.843, *p* < 0.001) with the density of *R. solanacearum* determined by qPCR (Supplementary Figure S5). This indicates that the abundance of the *Ralstonia* genus can represent the density of the pathogen *R. solanacearum*. In addition to *Ralstonia*, the abundances of *Ochrobactrum* and *Kluyvera* were also higher in the conducive soil than in the suppressive soil (Supplementary Figure S4A). In the fungal community, the *Fusarium* genus, which contains the fungal wilt pathogens in plants, was the most dominant group in the conducive soil and infected root (Figure 3D; Supplementary Figure S4B), accounting for 15.0 and 21.6% of the total fungal sequences, respectively (only 0.6 and 1.7% in the suppressive soil and healthy root, respectively). Interestingly, the relative abundance of *Fusarium* positively correlated with *Ralstonia* (Supplementary Figure S6), suggesting that bacterial wilt disease is probably accompanied by *Fusarium* wilt disease.

### Network Analysis of Bacterial and Fungal Communities

**Figure 4:**
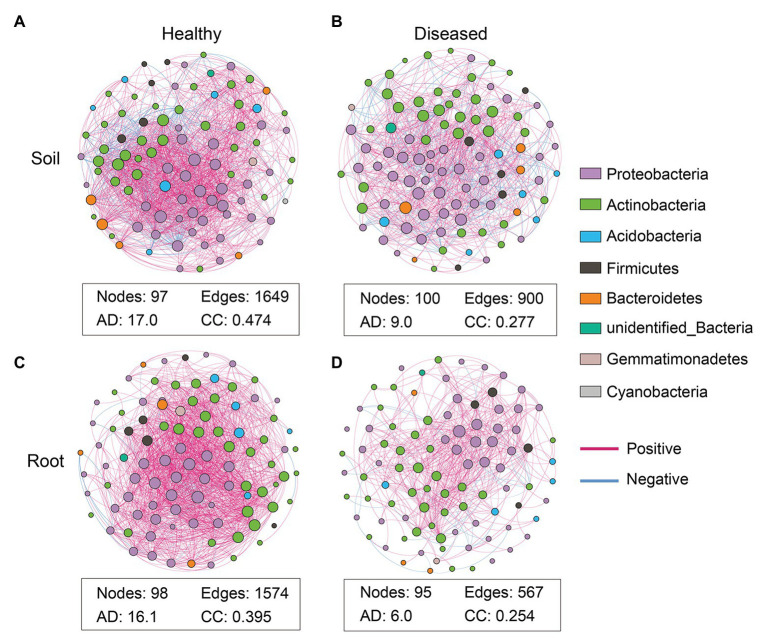
Co-occurrence network analyses of the rhizosphere soil and root based on top 100 abundant bacterial genera. Soil networks are shown in Panels (**A**; suppressive soil) and (**B**; conducive soil). Root network are shown in Panels (**C**; healthy root) and (**D**; infected root). The nodes in these networks represent genera and the edges represent correlations between the nodes. AD, average degree; the average number of direct correlations to a node in the network; CC, clustering coefficient. The probability that the adjacent nodes of a node are connected. A connection indicates a statistically significant [false discovery rate (FDR) adjusted *p* < 0.05] correlation with magnitude >0.6 (positive correlation: blue edges) or <−0.6 (negative correlation: red edges). The size of each node is proportional to the connection numbers of node (i.e., degree). Each node color represents a bacterial genus at phylum level.

To explore the microbial co-occurrence and potential keystone taxa in the suppressive soil and healthy root samples, network analyses were carried out based on the top 100 abundant genera. The networks showed remarkable differences in their structure and topological properties (Figure 4; Supplementary Table S2). For the bacterial community, the suppressive and conducive soils consisted of 97 and 100 nodes with a modularity of 0.444 and 0.731, with average path lengths of 2.343 and 3.125, respectively (Supplementary Table S2). Moreover, the suppressive soil harbored higher values of edges (total of 1,649: positive, 1,449; negative, 200), clustering coefficient (0.474), and average degree (17.0) than the conducive soil, which presented values of 900 (positive, 701; negative, 199), 0.277, and 9.0, respectively (Figures 4A,B; Supplementary Table S2). Similarly, the healthy roots also had a higher number of edges, clustering coefficient, average degree, lower modularity, and shorter average path length compared to the infected roots (Figures 4D,E; Supplementary Table S2). The above results revealed that the bacteria in the suppressive soil and healthy roots formed a highly interactive and complex network. Moreover, the infected root samples harbored a much simpler network than the conducive soil, suggesting that bacterial wilt disease may have a greater influence on the endophytic microbiome. Similar to the general community analysis, the phyla Proteobacteria, Actinobacteria, and Firmicutes seem to play a key role in the establishment of the microbiota sociability (Figure 4).

With respect to fungi, networks of the top 100 abundant genera were also constructed (Supplementary Figure S7; Supplementary Table S3). In contrast to the bacterial network, the conducive soil had higher edges (425), average degree (4.5), and clustering coefficient (0.203) than those (352, 3.8, and 0.189, respectively) in the suppressive soil. The healthy root samples had higher edges (418), average degree (5.1), and clustering coefficient (0.218) than those (331, 3.9, and 0.162, respectively) in the infected root samples. This indicated that the healthy roots possessed a more complex network compared to the infected roots, which was consistent with the bacterial network.

### Keystone Taxa Associated With Bacterial and Fungal Communities

**Figure 5:**
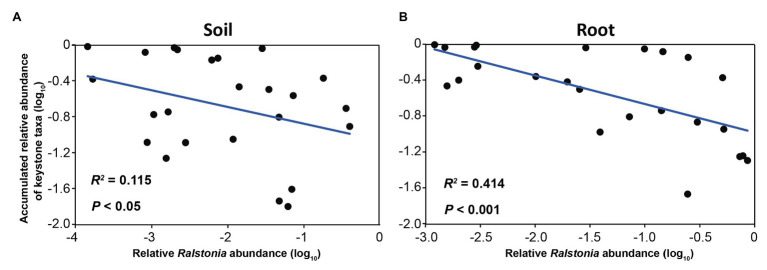
Relationship between the relative abundance of *Ralstonia* and the accumulated abundances of keystone taxa in **(A)** soil and **(B)** root.

To investigate the driving taxa behind the disease-suppressive soil and healthy root samples, the taxa with the high degree, high closeness centrality, and low betweenness centrality were calculated. The results showed that no bacterial keystone taxon was found in the conducive soils and infected roots, while 9 and 13 bacterial keystone taxa were obtained in the suppressive soil and healthy roots, respectively (Supplementary Table S4). Three and 10 fungal keystone taxa were found in the suppressive soil and healthy roots, respectively (Supplementary Table S5). Most of the bacterial keystone taxa were from Proteobacteria and Actinobacteria. Among all bacterial keystone taxa, *Pseudomonas* was the most abundant genus in both the suppressive soil and healthy roots, followed by *Streptomyces* and *Gaiella* (Supplementary Table S4). More importantly, the accumulated abundance of all bacterial keystone taxa negatively correlated with *Ralstonia* in the soil (*R*^2^ = 0.115, *p* < 0.05; Figure 5A) and root (*R*^2^ = 0.414, *p* < 0.001; Figure 5B), implying their potential beneficial effects on disease control. No correlation between fungal keystone taxa and *Ralstonia* was observed. NetShift analysis was carried out to identify drivers in the conducive soil and infected root samples (Supplementary Figure S8). A total of 24 and 25 bacterial genera whose betweenness (importance) increased from the suppressive soil to the conducive soil and from the healthy root to the infected root samples, respectively, were identified as potential driver taxa. Among these taxa, 13 taxa were shared by the conducive soil and infected roots. *Ralstonia*, *Kluyvera*, *Terrabacter*, *Ochrobactrum*, and *Massilia* were considered to play significant roles in maintaining the network structure of the conducive soil and infected roots.

### Inhibitory Effects of Isolates on *R. solanacearum* and Greenhouse Experiments

**Figure 6:**
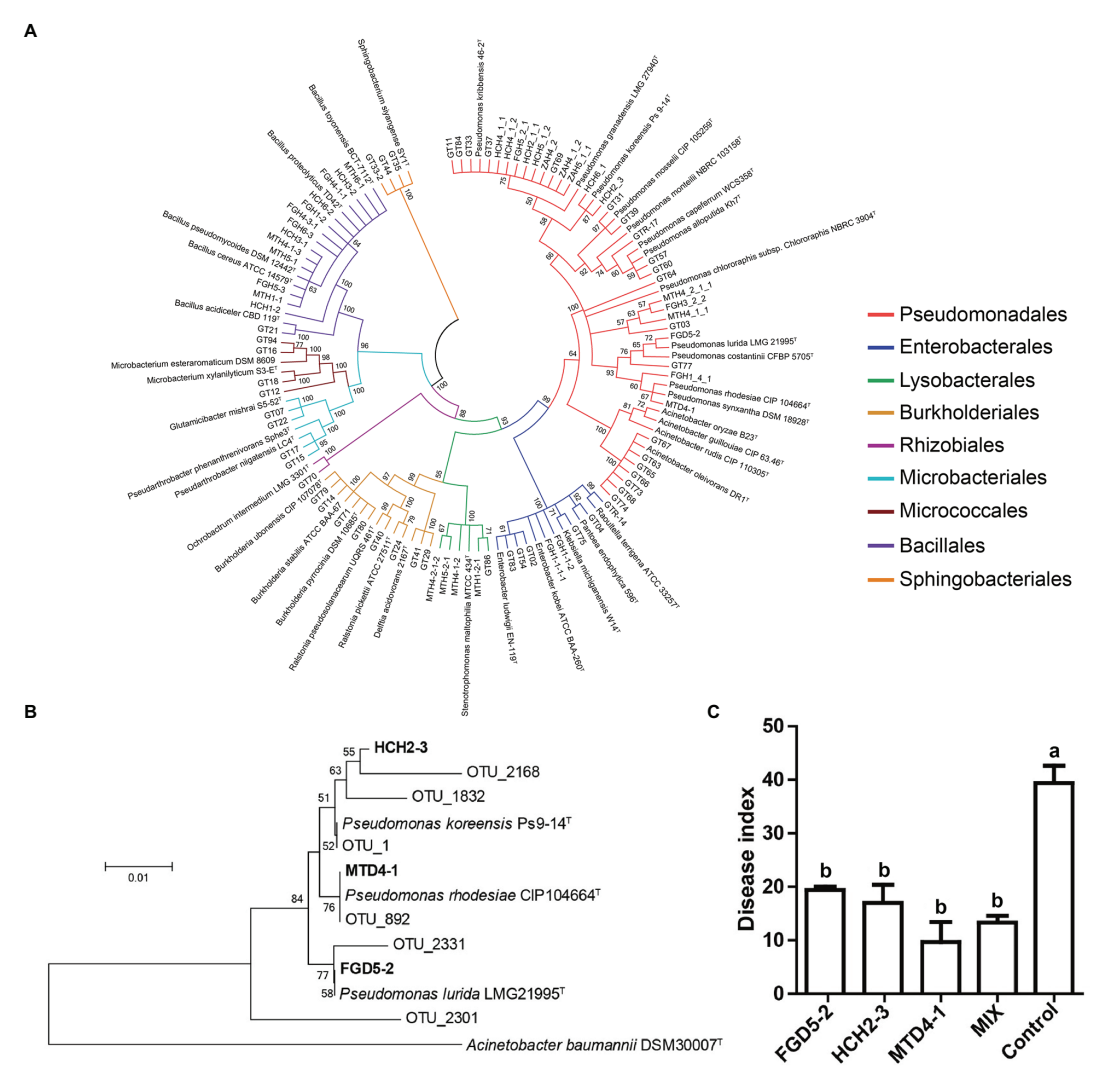
The phylogenetic and disease suppression analyses of potential keystone bacteria. **(A)** The neighbor-joining phylogenetic tree based on 16S rRNA gene sequences of 83 culturable isolates and their type strain. **(B)** The neighbor-joining phylogenetic tree constructed with the 16S rRNA gene sequences obtained from high-throughput amplicon sequencing derived operational taxonomic units (OTUs) and the isolated strains. **(C)** The disease index of tobacco seedlings after *R. solanacearum* inoculation for 15 days. Bars (the standard error of the mean) represent the average of three biological replicates (*n* = 12) per treatment. Different letters above the bars in Panel **(C)** indicate a significant difference (*p* < 0.05) according to the Tukey’s test.

To complement the network analysis and further investigate the important role keystone taxa might play in suppressing disease, we performed an isolation campaign and isolated 83 bacterial strains from a healthy root sample. These root-derived isolates represented four bacterial phyla and nine bacterial orders (Figure 6A). Unfortunately, only *Pseudomonas* sp. of the keystone taxa was obtained. Three *Pseudomonas* isolates, *P. lurida* FGD5-2, *P. koreensis* HCH2-3, and *P. rhodesiae* MTD4-1 showed 98.4–100% of 16S rRNA gene similarity to *Pseudomonas* OTU sequences derived from high-throughput amplicon sequencing and clustered together (Figure 6B), indicating that these strains represent indigenous key species of healthy plants. Thus, they were selected for further experiment. We found that three *Pseudomonas* isolates displayed strong inhibitory effects on *R. solanacearum*, with an inhibition zone ranging from 2.9 to 4.2 cm (Supplementary Table S6). Moreover, greenhouse experiments were performed to determine whether they could protect plants against *R. solanacearum* infection. The results showed that all *Pseudomonas* addition groups could significantly reduce disease indices (Tukey’s test, *p* < 0.05) when compared to the control group (Figure 6C). The consortium consisted of three *Pseudomonas* isolates, thereby showing similar protection against disease with strain MTD4-1. Overall, this indicated that *Pseudomonas* species (one of the keystone taxa) might play a significant role in maintaining tobacco health while decreasing bacterial wilt incidence.

## Discussion

In this study, we observed that in two adjacent tobacco experimental fields, plants in conducive soil suffered severe wilt disease, but in suppressive soil, they grew well. Our objective was to find microbial keystone taxa behind this phenomenon using network analysis and explore their relationship with bacterial wilt pathogen. We found that both bacterial and fungal network complexity between the suppressive and conducive soils were different. Several keystone taxa were identified from the suppressive soil. Importantly, we further found that isolates presumably related to keystone taxa have inhibitory effects on the pathogen and reduction of bacterial wilt disease incidence, highlighting the importance of keystone taxa in suppressing disease.

Large numbers of previous studies have analyzed the soil microbial community, abundance, and diversity when bacterial wilt disease outbreak (Wang et al., 2017b; Qi et al., 2019; Wei et al., 2019; Lee et al., 2020). As they reported, we also found that the relative abundance of Firmicutes was higher in the suppressive soil compared to that in the conducive soil (Figure 3A). The phylum Firmicutes is well known to include species that produce various secondary metabolites beneficial to plant growth (Kim et al., 2011), and disruption of its abundance in tomato rhizosphere causes the incidence of bacterial wilt disease (Lee et al., 2020). In the fungal community, Ascomycota and Basidiomycota were the most abundant phyla in all the samples (Figure 3B), broadly corresponding to extensive surveys of the soil microbiome (Hartmann et al., 2015; Fu et al., 2017; Xiong et al., 2017). In this study, we found that bacterial wilt disease causes the reduction in Ascomycota and enrichment of Basidiomycota.

Consistent with field observations of the plants, OTUs assigned to the potential pathogenic *Ralstonia* showed high abundances in the conducive soil and infected roots but were rarely found in the suppressive soil and healthy roots (Figure 3C). In the fungal community, the relative abundances of *Fusarium* in the conducive soil and infected roots were 25- and 13-fold higher than those in the suppressive soil and healthy roots, respectively (Figure 3D), indicating that the occurrence of bacterial wilt disease might be accompanied by a high abundance of *Fusarium*. This is supported by the positive correlation between the abundance of *Fusarium* and *Ralstonia*.

It has been suggested that the function of a plant microbiome is not the sum of its individual members, since microbial taxa can interact with each other and shape a complex network (van der Heijden and Hartmann, 2016). Our study revealed that disease altered the network features of the soil and root microbiomes. In particular, the co-occurrence networks of the suppressive soil and healthy roots were more complex, with a higher number of edges, higher average degree, and clustering coefficient compared with the networks of the conducive soil and infected roots (Figure 4); this is consistent with previous studies (Qi et al., 2019; Wei et al., 2019; Zhang et al., 2020). It was indicated that complex networks are more robust to external biotic and abiotic stresses than simple networks (Santolini and Barabási, 2018). Thus, the high-complexity network of the suppressive soil and healthy root in this study might be critical for the suppression of disease. The key members of microbial communities are defined as keystone species, which frequently interact with other microbial taxa in co-occurrence networks. In this study, no keystone taxon was observed in the conducive soil and infected roots, while 9 and 13 keystone taxa, most of them belonging to Proteobacteria and Actinobacteria, were identified in the suppressive soil and healthy roots, respectively (Supplementary Table S4). The high abundance and diversity of these keystone taxa can increase the complexity of healthy plant networks, leading to a highly resilient microbiome. It has been found that keystone taxa play a determinant role within the microbiome, and their removal causes significant changes in network complexity, microbial composition, and function (Banerjee et al., 2018, 2019). Furthermore, the accumulated abundance of all keystone taxa exhibited a negative correlation with the abundance of *Ralstonia* (Figure 5), indicating the importance of their roles in suppressing bacterial wilt disease.

Keystones identified by network-based scores must be complemented with experimental evidence to uncover their true importance (Banerjee et al., 2018). Therefore, we performed an isolation campaign to obtain culturable keystone taxa. Unfortunately, only species of the *Pseudomonas* genus within the keystone taxa were obtained in our study. By constructing a phylogenetic tree with sequences of OTUs and isolates, three *Pseudomonas* species were considered as indigenous strains, and all of them showed strong inhibitory effects on *R. solanacearum* plates. Furthermore, a greenhouse experiment suggested that these *Pseudomonas* species could significantly reduce the disease indices of tobacco plants (Figure 6C). Consistently, Hu et al. (2016) found that diverse *Pseudomonas* consortia enhanced rhizosphere microbiome function and plant disease suppression. We know that numerous *Pseudomonas* species have been found from suppressive soil and are used as protectants against soil-borne diseases (Schroth and Hancock, 1982; Keel et al., 1992; Haas and Keel, 2003). However, to our knowledge, this is the first study to reveal their indispensable role as keystone taxa in sustaining the stability of healthy plant microbiomes. Many other keystone taxa, such as *Streptomyces* (Tan et al., 2011), *Ensifer* (Kumar et al., 2011), *Bradyrhizobium* (Omar and Abd-Alla, 1998), and *Microbacterium* (Freitas et al., 2019) have been reported to be beneficial for plant growth and/or antagonistic to *R. solanacearum*.

**Figure 7:**
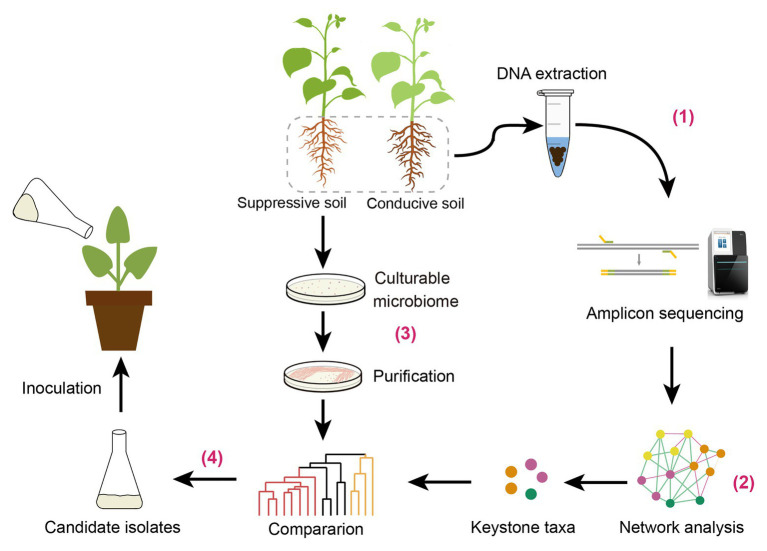
The strategy we proposed for biocontrol agent exploring based on rhizosphere keystone taxa.

It has been indicated that most exogenous microorganisms cannot colonize in plant rhizosphere under field conditions (Gómez Expósito et al., 2017). Recently, synthetic community (SynCom) comprising multiple microbes has been constructed in sterile soil to imitate disease-suppressive community (Bai et al., 2015; Liu et al., 2019). Identification of keystone taxa from disease-suppressive soil may provide insights to design an artificial SynCom against soil-borne diseases. In this study, we identified keystone taxa through network analysis at the genus level and confirmed their preventive effect on disease suppression. Based on the results of previous and current studies, we propose a new strategy for biocontrol agent exploring based on rhizosphere core microbiome. The potential strategy may include the following steps (Figure 7): (1) rhizosphere microbiome analysis through high-throughput sequencing techniques; (2) screening microbial keystone taxa associated to plant health by network analysis; (3) the cultivable biocontrol agents screening; and (4) select candidate agent by comparison to the microbial keystone taxa. It should be noted that the biocontrol effect of these strains selected should be further verified by field experiments.

## Conclusion

The suppressive and conducive soils showed substantial shifts in microbial abundance and community composition. High abundance of *Fusarium* in the conducive soil and infected roots suggests that *R. solanacearum* infection probably boosts the growth of *Fusarium* within the fungal community. The network complexity and abundance of keystone taxa are higher in the suppressive soil than in the conducive soil. A negative association was observed between the accumulated abundance of keystone taxa and the abundance of *Ralstonia*. The genus *Pseudomonas* was the most abundant keystone taxon, and greenhouse experiments showed that several species in this genus could reduce the disease index. Thus, we propose that the keystone species in suppressive soil and/or healthy plants play an important role in the suppression of soil-borne pathogens.

## Data Availability Statement

The datasets presented in this study can be found in online repositories. The names of the repository/repositories and accession number(s) can be found below: https://www.ncbi.nlm.nih.gov/, PRJNA632641.

## Author Contributions

C-SZ and ML designed the experiments, analyzed the data, and wrote the manuscript. YZ conducted qPCR, analyzed the sequence data, wrote the manuscript, and prepared all figures and tables. XH collected samples and analyzed data. DZ and KW extracted community DNA and isolated strains. KW performed greenhouse experiment. YY and YL discussed the results and provide critical idea in greenhouse experiment. All authors contributed to the article and approved the submitted version.

### Conflict of Interest

The authors declare that the research was conducted in the absence of any commercial or financial relationships that could be construed as a potential conflict of interest.

## Footnotes

^1^https://web.rniapps.net/netshift/
