# Supplementary material for: Exploring Biocontrol Agents From Microbial Keystone Taxa Associated to Suppressive Soil: A New Attempt for a Biocontrol Strategy
Source: Front Plant Sci. 2021 Mar 19;12:655673. doi: 10.3389/fpls.2021.655673 (PMC8095248; doi:10.3389/fpls.2021.655673)
Supplement: Supplementary file 1 [file Data_Sheet_1.docx]

**Supplementary material for**

**Exploring biocontrol agents from microbial keystone taxa associated to suppressive soil: a new attempt for a biocontrol strategy**

Yanfen Zheng^1†^, Xiaobin Han^2†^, Donglin Zhao^1^, Keke Wei^1^, Yuan Yuan^1^, Yiqiang Li^1^, Minghong Liu^2*^, Cheng-Sheng Zhang^1*^

^1^Pest Integrated Management Key Laboratory of China Tobacco, Tobacco Research Institute of Chinese Academy of Agricultural Sciences, Qingdao, China

^2^Biological Organic Fertilizer Engineering Technology Center of China Tobacco, Zunyi Branch of Guizhou Tobacco Company, Zunyi, China

^†^These authors contributed equally to this work

^*^Correspondence: zhangchengsheng@caas.cn; lmh859@163.com

**Figure S1 Bacterial and *R. solanacearum* pathogen abundances determined by qPCR.** Statistical analysis of the data was performed using Student’s *t*-test. ***P* < 0.01; **P* < 0.05. Bars represent the standard error of the mean.

**Figure S2 Rarefaction curve showing the number of bacterial and fungal OTUs plotted against number of sequences.** SS: suppressive rhizosphere soil; CS: conducive rhizosphere soil; HR: root of healthy tobacco; IR: root of infected tobacco.

**Figure S3 Venn** [**diagram**](javascript:;)**of bacterial and fungal communities at the OTU level.** SS: suppressive rhizosphere soil; CS: conducive rhizosphere soil; HR: root of healthy tobacco; IR: root of infected tobacco.

**Figure S4 Bacterial (a) and fungal (b) communities at the genus level showed significant difference (*P* < 0.05) between suppressive and diseased samples by *t*-test**. SS: suppressive rhizosphere soil; CS: conducive rhizosphere soil; HR: root of healthy tobacco; IR: root of infected tobacco.

**Figure S5 Relationship between the relative abundance of *Ralstonia* determined by high throughput sequencing and the absolute abundance of** ***R. solanacearum* determined by qPCR.**

**Figure S6** **Relationship between the relative abundances of** ***Ralstonia* and *Fusarium*.**

**Figure S7.** **Co-occurrence network analyses of the rhizosphere soil and root based on top 100 abundant fungal genera.** Soil network are shown in **a** (healthy sample) and **b** (diseased sample). Root network are shown in **c** (healthy sample) and **d** (diseased sample). The nodes in these networks represent genera and the edges represent correlations between the nodes. AD: average degree; the average number of direct correlations to a node in the network. CC: clustering coefficient; the probability that the adjacent nodes of a node are connected. A connection stands for a statistically significant (FDR adjusted *P* < 0.05) correlation with magnitude > 0.6 (positive correlation: blue edges) or < -0.6 (negative correlation: red edges). The size of each node is proportional to the connection numbers of node (i.e. degree). Each node color represents a fungal genus at phylum level.

**Figure S8 The potential driver taxa for changes observed in bacterial co-occurrence networks between healthy and diseased samples.** Node sizes are proportional to their scaled NESH score (a score identifying the importance of given microbial taxa in the association network). The node colored in red means its betweenness (importance) increased from healthy to diseased samples. Hence, nodes which are big and red are particularly important 'drivers'. Red words represent the driver taxa present in both (soil and root).

**Table S1 Primers used in this study.**

| Name | Sequences (5’-3’) | Annealing temperature | Usage | Reference |
| --- | --- | --- | --- | --- |
| 799F | AACMGGATTAGATACCCKG | 55 | 16S rRNA gene amplicon sequencing | Chelius and Triplett, 2001 |
| 1193R | ACGTCATCCCCACCTTCC |  |  | Bodenhausen, et al., 2013 |
| ITS1F | CTTGGTCATTTAGAGGAAGTAA | 57 | ITS amplicon sequencing | Adams, et al., 2013 |
| ITS2R | GCTGCGTTCTTCATCGATGC |  |  |  |
| Eub338 | ACTCCTACGGGAGGCAGCAG | 55 | qPCR for bacteria | Muyzer, et al., 1993 |
| Eub518 | ATTACCGCGGCTGCTGG |  |  |  |
| Rsol_*fliC*f | GAACGCCAACGGTGCGAACT | 60 | qPCR for *Ralstonia* | Schonfeld, et al., 2003 |
| Rsol_*fliC*r | GGCGGCCTTCAGGGAGGTC |  |  |  |
| 27F | AGAGTTTGATCCTGGCTCAG | 55 | Isolates identification | Weisburg, et al., 1991 |
| 1492R | GGTTACCTTGTTACGACTT |  |  |  |

**Table S2 The network properties of bacterial communities in different samples.** SS: suppressive rhizosphere soil; CS: conducive rhizosphere soil; HR: root of healthy tobacco; IR: root of infected tobacco.

| **Samples** | **Modularity**  **(MD)** | **Average path length (APL)** | **Positive edges** | **Negative edges** |
| --- | --- | --- | --- | --- |
| **SS** | 0.444 | 2.343 | 1,449 | 200 |
| **CS** | 0.731 | 3.125 | 701 | 199 |
| **HR** | 0.272 | 2.281 | 1,493 | 81 |
| **IR** | 0.350 | 3.356 | 534 | 33 |

**Table S3 The network properties of fungal communities in different samples.** SS: suppressive rhizosphere soil; CS: conducive rhizosphere soil; HR: root of healthy tobacco; IR: root of infected tobacco.

| **Samples** | **Modularity**  **(MD)** | **Average path length (APL)** | **Positive edges** | **Negative edges** |
| --- | --- | --- | --- | --- |
| **SS** | 0.537 | 3.836 | 329 | 23 |
| **CS** | 0.810 | 3.093 | 338 | 87 |
| **HR** | 0.332 | 4.034 | 391 | 27 |
| **IR** | 0.747 | 3.184 | 259 | 72 |

**Table S4. Bacterial keystone taxa in suppressive soil and healthy root samples.** Keystones were defined on the basis of high degree, high closeness centrality (CC) and low betweenness centrality (BC) with cut-off value of degree > 40 and CC > 0.61 and BC < 0.18 according to Berry and Widder (2014) (Berry and Widder, 2014).

| **Genus** | **Abundance**  **(%)** | **Phylum** | **Degree** | **Closeness**  **centrality** | **Betweenness**  **centrality** |
| --- | --- | --- | --- | --- | --- |
| **Rhizosphere soil** |  |  |  |  |  |
| *Ensifer* | 0.38 | Proteobacteria | 50 | 0.66 | 0.116 |
| *Nordella* | 0.03 | Proteobacteria | 48 | 0.65 | 0.115 |
| *Pseudomonas* | 45.90 | Proteobacteria | 45 | 0.63 | 0.083 |
| *Gaiella* | 0.99 | Actinobacteria | 46 | 0.62 | 0.067 |
| *Roseateles* | 0.03 | Proteobacteria | 48 | 0.62 | 0.103 |
| *Blastococcus* | 0.41 | Actinobacteria | 43 | 0.62 | 0.085 |
| Unclassified_Acidobacteria | 0.11 | Acidobacteria | 40 | 0.61 | 0.056 |
| *Variovorax* | 0.22 | Proteobacteria | 44 | 0.61 | 0.112 |
| *Methylobacillus* | 0.17 | Proteobacteria | 46 | 0.61 | 0.127 |
| **Root** |  |  |  |  |  |
| *Pseudomonas* | 52.53 | Proteobacteria | 59 | 0.66 | 0.171 |
| *Rhizobacter* | 0.08 | Proteobacteria | 54 | 0.63 | 0.104 |
| *Conexibacter* | 0.02 | Actinobacteria | 53 | 0.63 | 0.075 |
| *Bradyrhizobium* | 0.32 | Proteobacteria | 52 | 0.63 | 0.082 |
| *Mycobacterium* | 2.02 | Actinobacteria | 51 | 0.62 | 0.071 |
| *Streptomyces* | 2.50 | Actinobacteria | 51 | 0.61 | 0.066 |
| *Lysinimonas* | 0.83 | Actinobacteria | 48 | 0.61 | 0.066 |
| *Bosea* | 0.37 | Proteobacteria | 49 | 0.61 | 0.041 |
| *Microbacterium* | 2.15 | Actinobacteria | 49 | 0.61 | 0.108 |
| *Devosia* | 0.92 | Proteobacteria | 48 | 0.61 | 0.035 |
| *Ensifer* | 2.12 | Proteobacteria | 48 | 0.61 | 0.049 |
| *Rhizorhapis* | 0.03 | Proteobacteria | 48 | 0.61 | 0.049 |
| *Nocardioides* | 0.67 | Actinobacteria | 48 | 0.61 | 0.079 |

**Table S5. Fungal keystone taxa in suppressive soil and healthy root samples.** Keystones were defined on the basis of high degree and high closeness centrality (CC) with cut-off value of degree > 20 and CC > 0.28.

| **Genus** | **Phylum** | **Degree** | **Closeness**  **centrality** |
| --- | --- | --- | --- |
| **Rhizosphere soil** |  |  |  |
| *Curvularia* | Ascomycota | 23 | 0.39 |
| *Acaulium* | Ascomycota | 21 | 0.53 |
| *Gibellulopsis* | Ascomycota | 20 | 0.38 |
| **Root** |  |  |  |
| *Emericellopsis* | Ascomycota | 30 | 0.37 |
| *Gibellulopsis* | Ascomycota | 29 | 0.29 |
| *Trichoderma* | Ascomycota | 28 | 0.29 |
| *Golovinomyces* | Ascomycota | 27 | 0.35 |
| *Phoma* | Ascomycota | 27 | 0.38 |
| *Curvularia* | Ascomycota | 27 | 0.38 |
| *Gamsia* | Ascomycota | 25 | 0.31 |
| *Moesziomyces* | Basidiomycota | 25 | 0.43 |
| *Gibberella* | Ascomycota | 22 | 0.31 |
| *Acremonium* | Ascomycota | 21 | 0.48 |

**Table S6. The inhibition zone diameters of strains having high similarity to keystone taxa**.

| **Strain No.** | **Closest species** | **Inhibition zone diameters (cm)** |
| --- | --- | --- |
| FGD5-2 | *Pseudomonas lurida* | 3.7 ± 0.10 |
| MTD4-1 | *Pseudomonas rhodesiae* | 2.9 ± 0.08 |
| HCH2-3 | *Pseudomonas koreensis* | 4.2 ± 0.15 |

**References**

Adams, R. I., Miletto, M., Taylor, J. W., and Bruns, T.D. (2013). Dispersal in microbes: fungi in indoor air are dominated by outdoor air and show dispersal limitation at short distances. *ISME J.* **7**, 1262-1273.

Berry, D., and Widder, S. (2014). Deciphering microbial interactions and detecting keystone species with co-occurrence networks. *Front Microbiol* **5**, 219.

Bodenhausen, N., Horton, M. W., and Bergelson, J. (2013). Bacterial communities associated with the leaves and the roots of *Arabidopsis thaliana*. *PLoS One* **8**, e56329.

Chelius, M. K., and Triplett, E. W. (2001). The Diversity of Archaea and Bacteria in Association with the Roots of *Zea mays* L. *Microb Ecol* **41**, 252-263.

Muyzer, G., de Waal, E. C., Uitterlinden, A. G. (1993). Profiling of complex microbial populations by denaturing gradient gel electrophoresis analysis of polymerase chain reaction-amplified genes coding for 16S rRNA. *Appl Environ Microbiol* **59**, 695-700.

Schonfeld, J., Heuer, H., Van Elsas, J. D., Smalla, K. (2003). Specific and sensitive detection of *Ralstonia solanacearum* in soil on the basis of PCR amplification of *fliC* fragments. *Appl Environ Microbiol* **69**, 7248-7256.

Weisburg, W. G., Barns, S. M., Pelletier, D. A., Lane, D. J. (1991). 16S ribosomal DNA amplification for phylogenetic study. J Bacteriol **173**, 697-703.
