# Supplementary figures and images for: Exploring Biocontrol Agents From Microbial Keystone Taxa Associated to Suppressive Soil: A New Attempt for a Biocontrol Strategy
Source: Front Plant Sci. 2021 Mar 19;12:655673. doi: 10.3389/fpls.2021.655673 (PMC8095248; doi:10.3389/fpls.2021.655673)

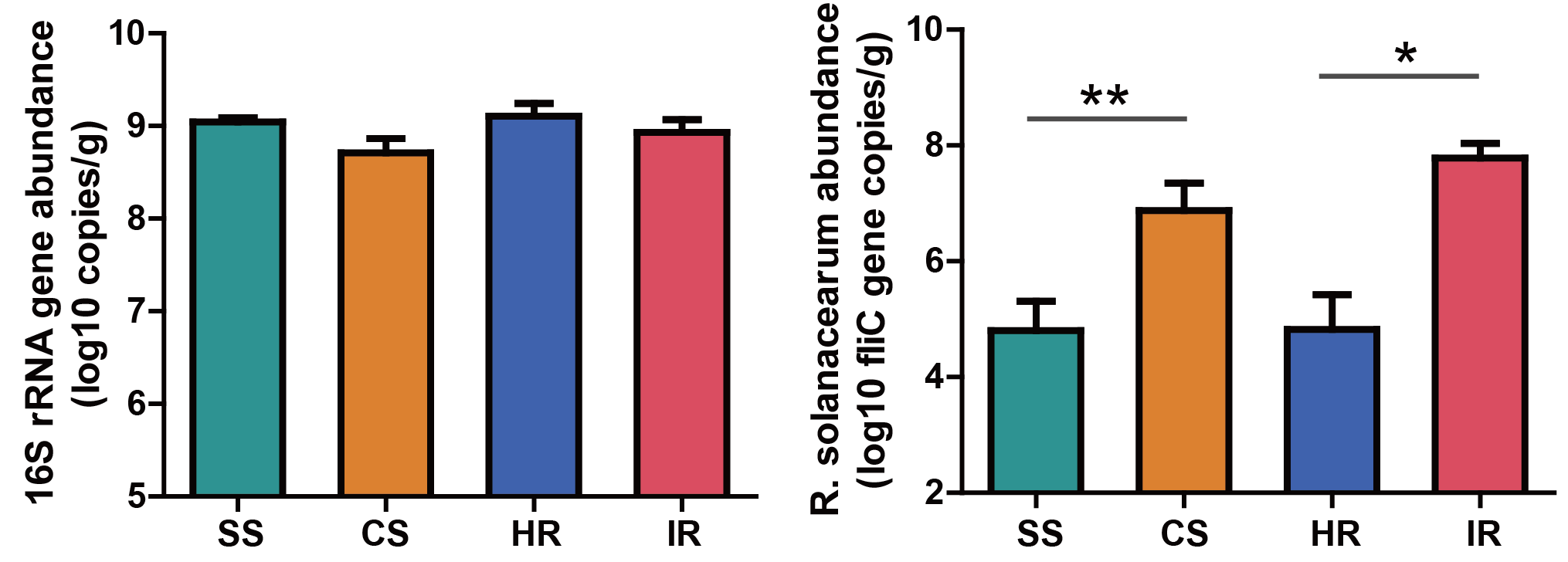

Supplement: Supplementary file 2 [file Image_1.tif]

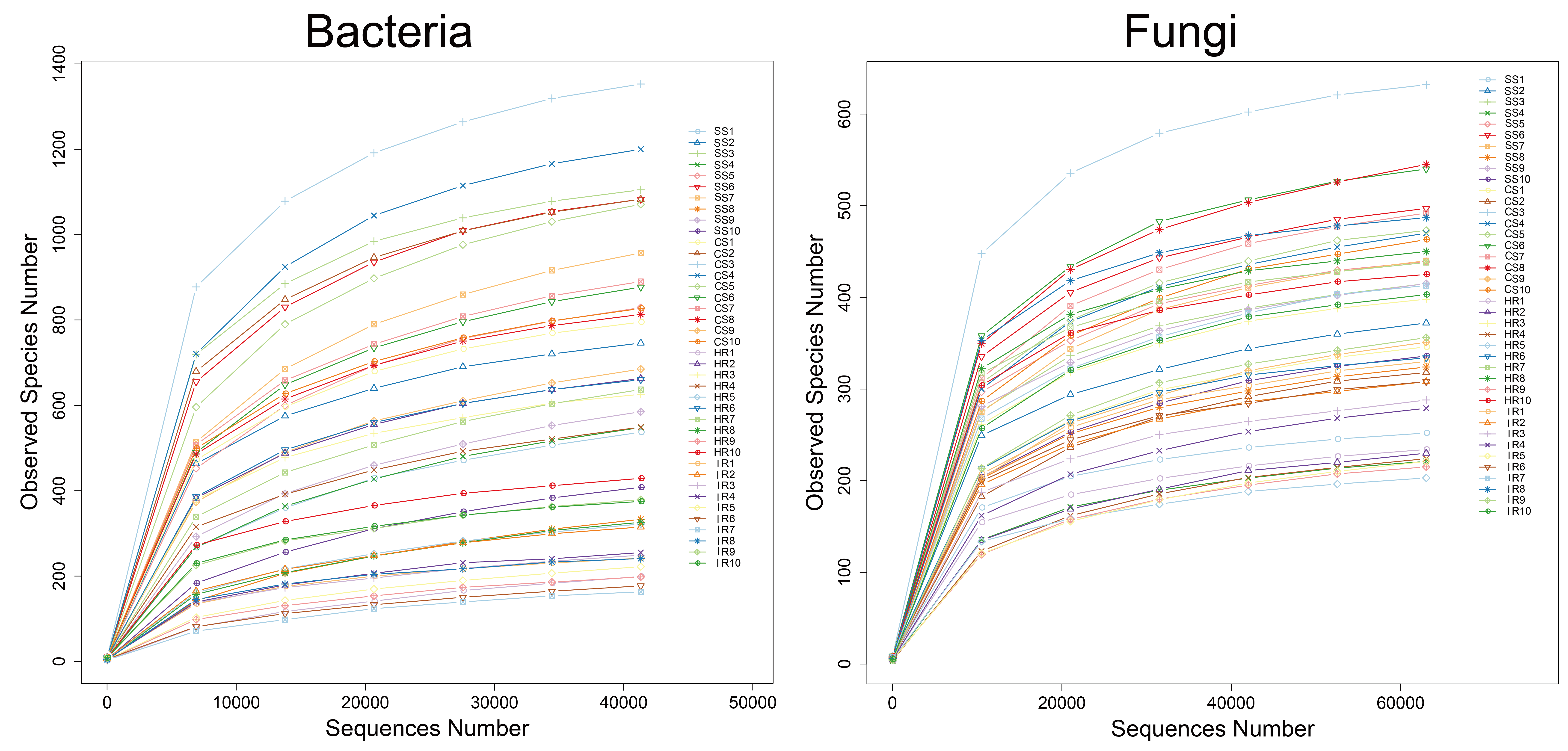

Supplement: Supplementary file 3 [file Image_2.tif]

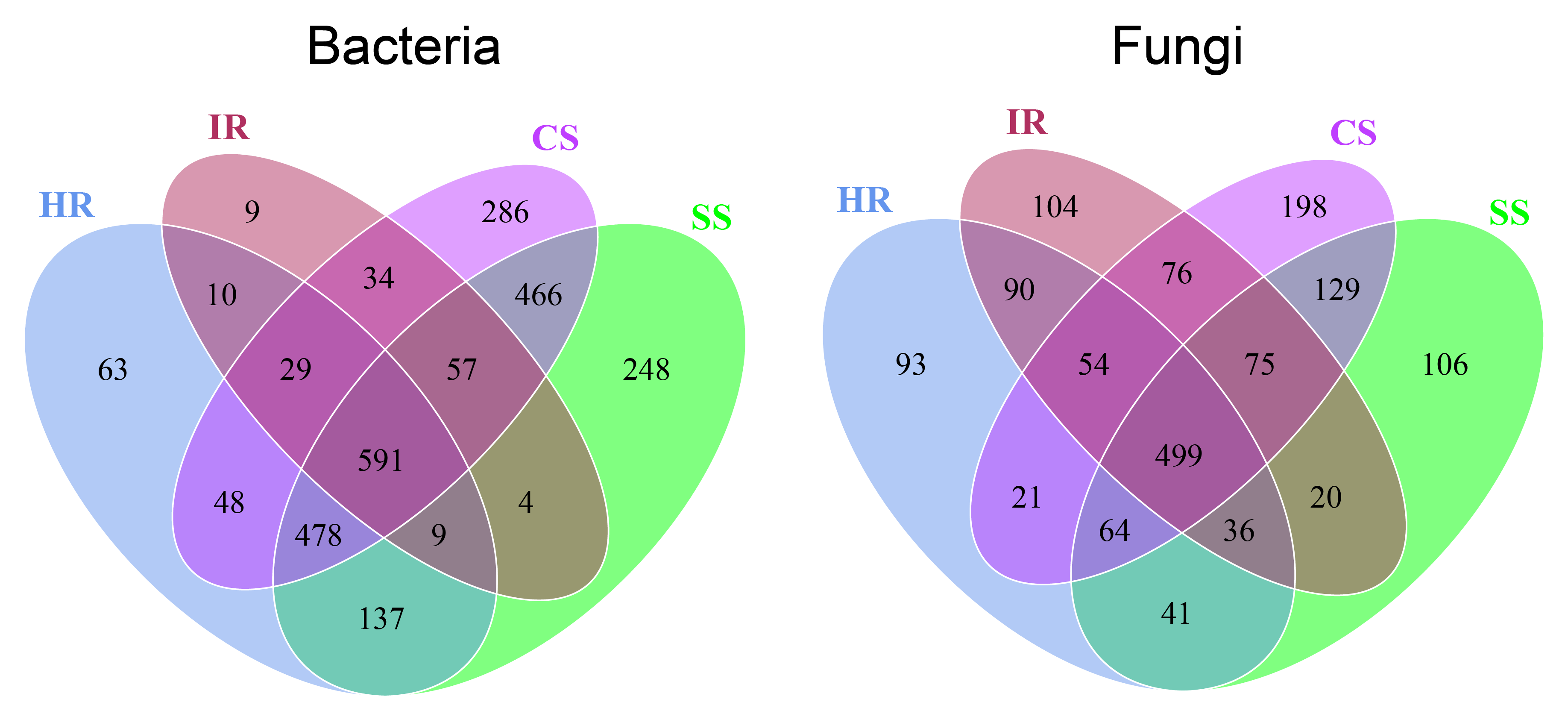

Supplement: Supplementary file 4 [file Image_3.tif]

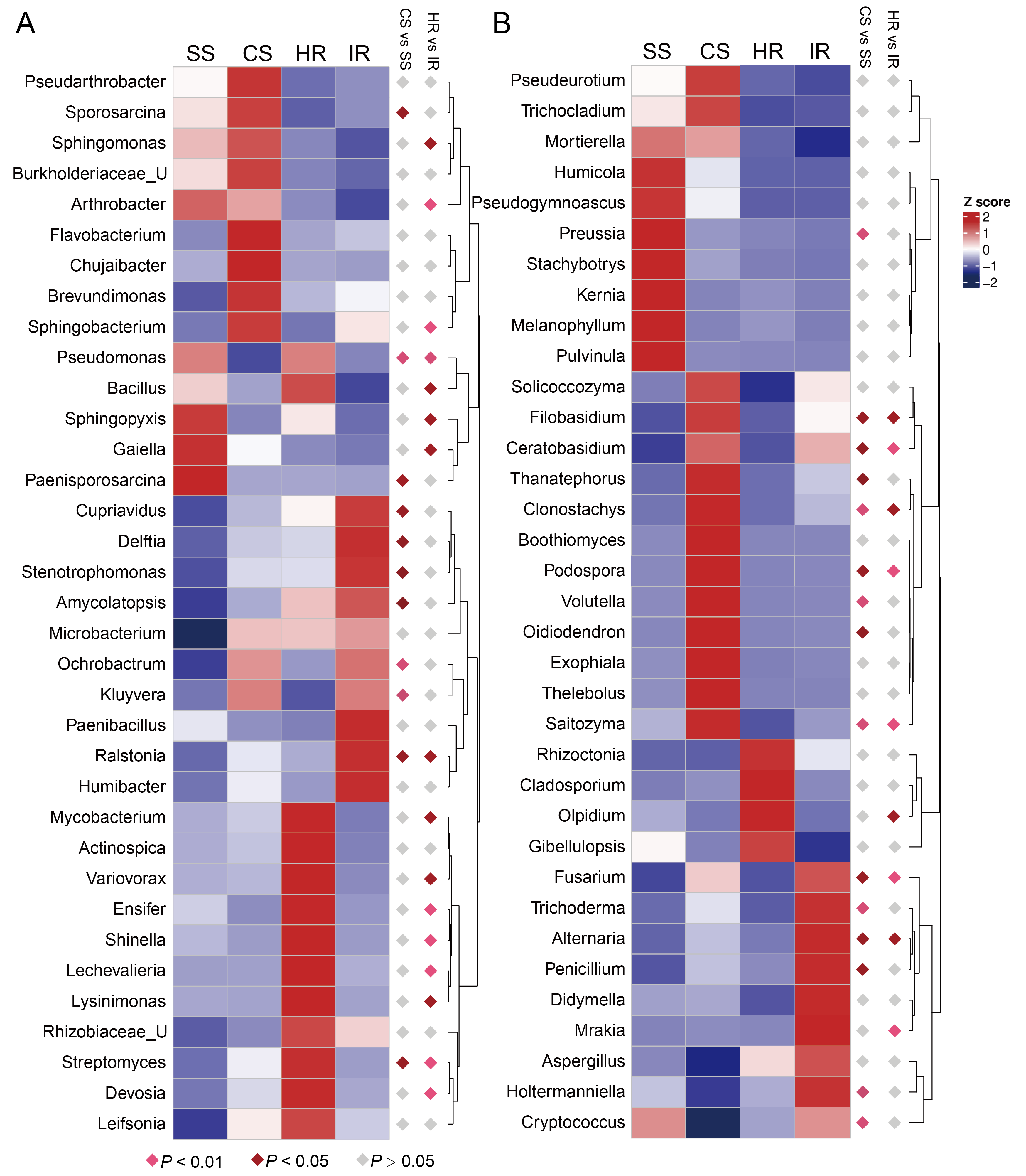

Supplement: Supplementary file 5 [file Image_4.tif]

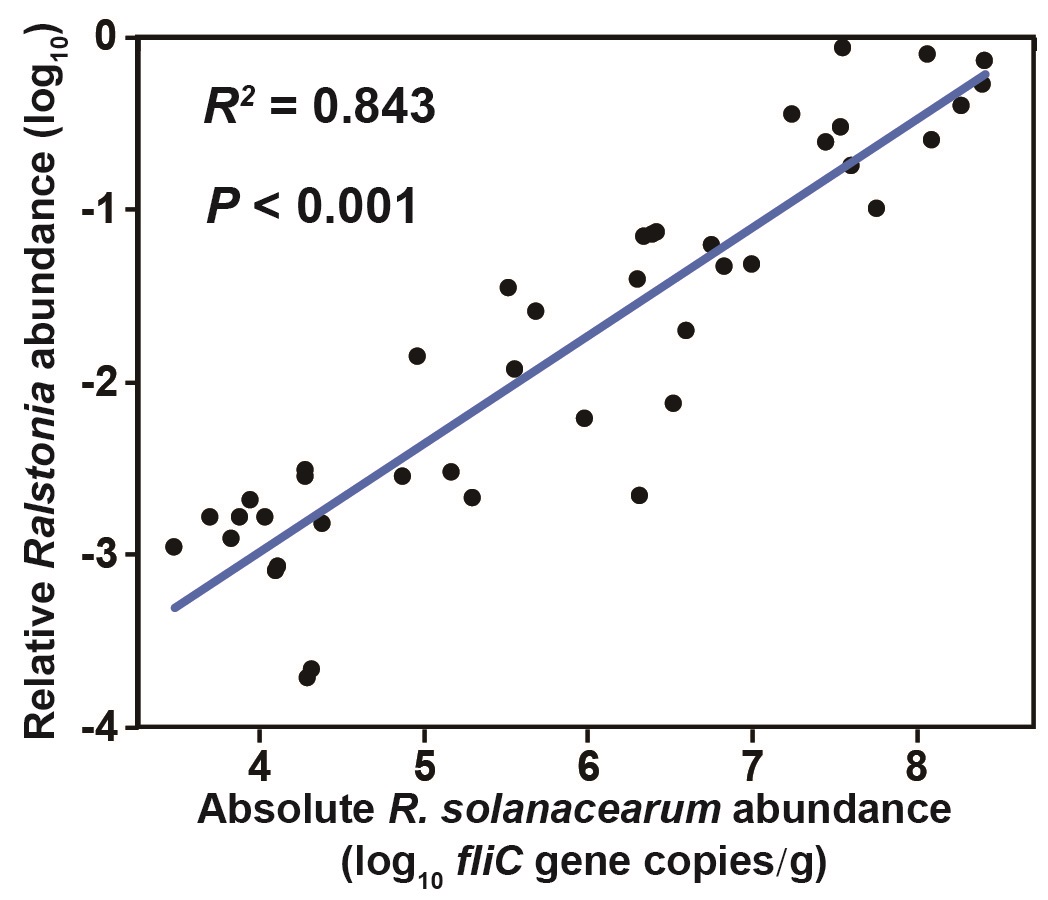

Supplement: Supplementary file 6 [file Image_5.jpeg]

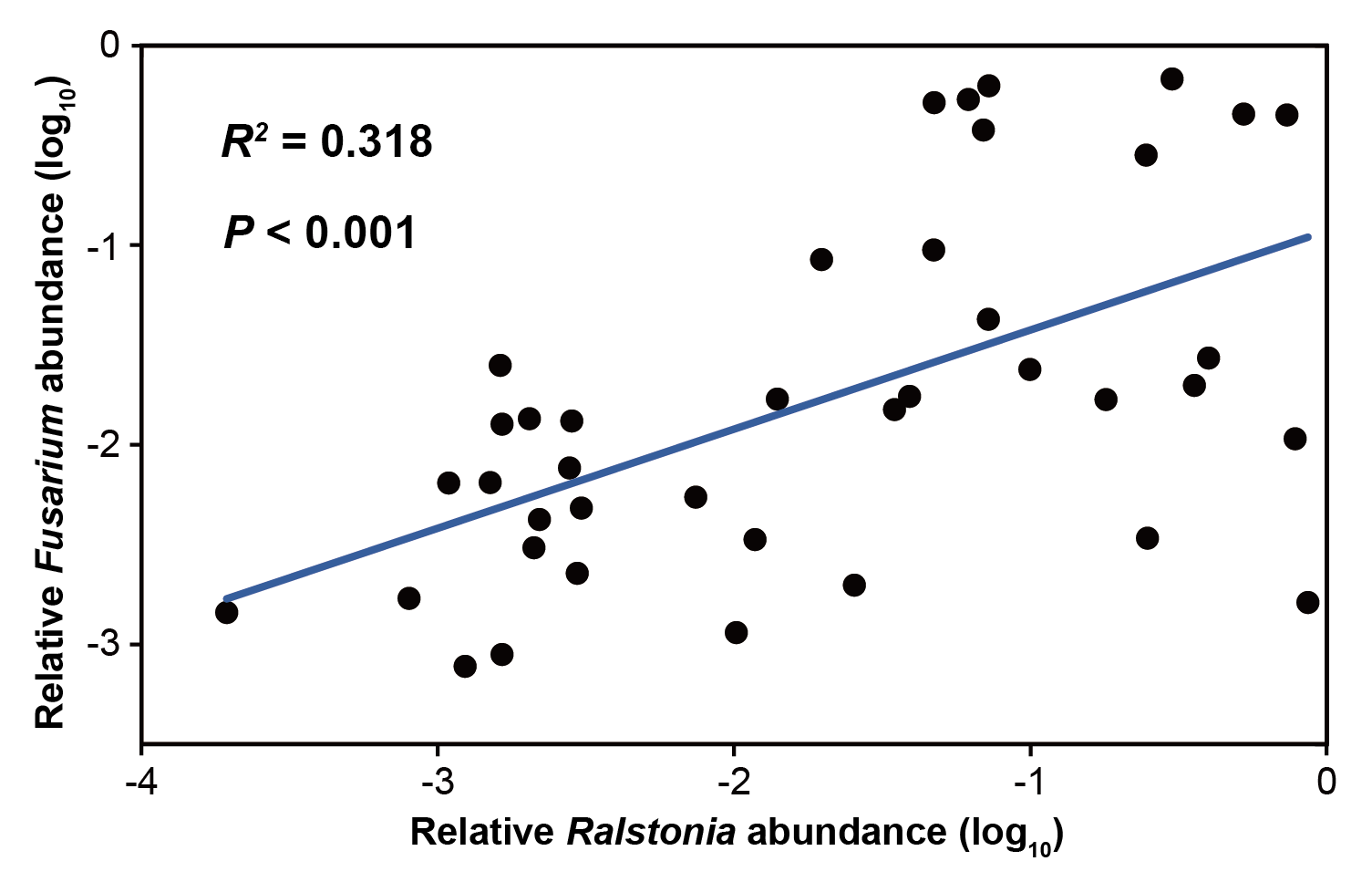

Supplement: Supplementary file 7 [file Image_6.tif]

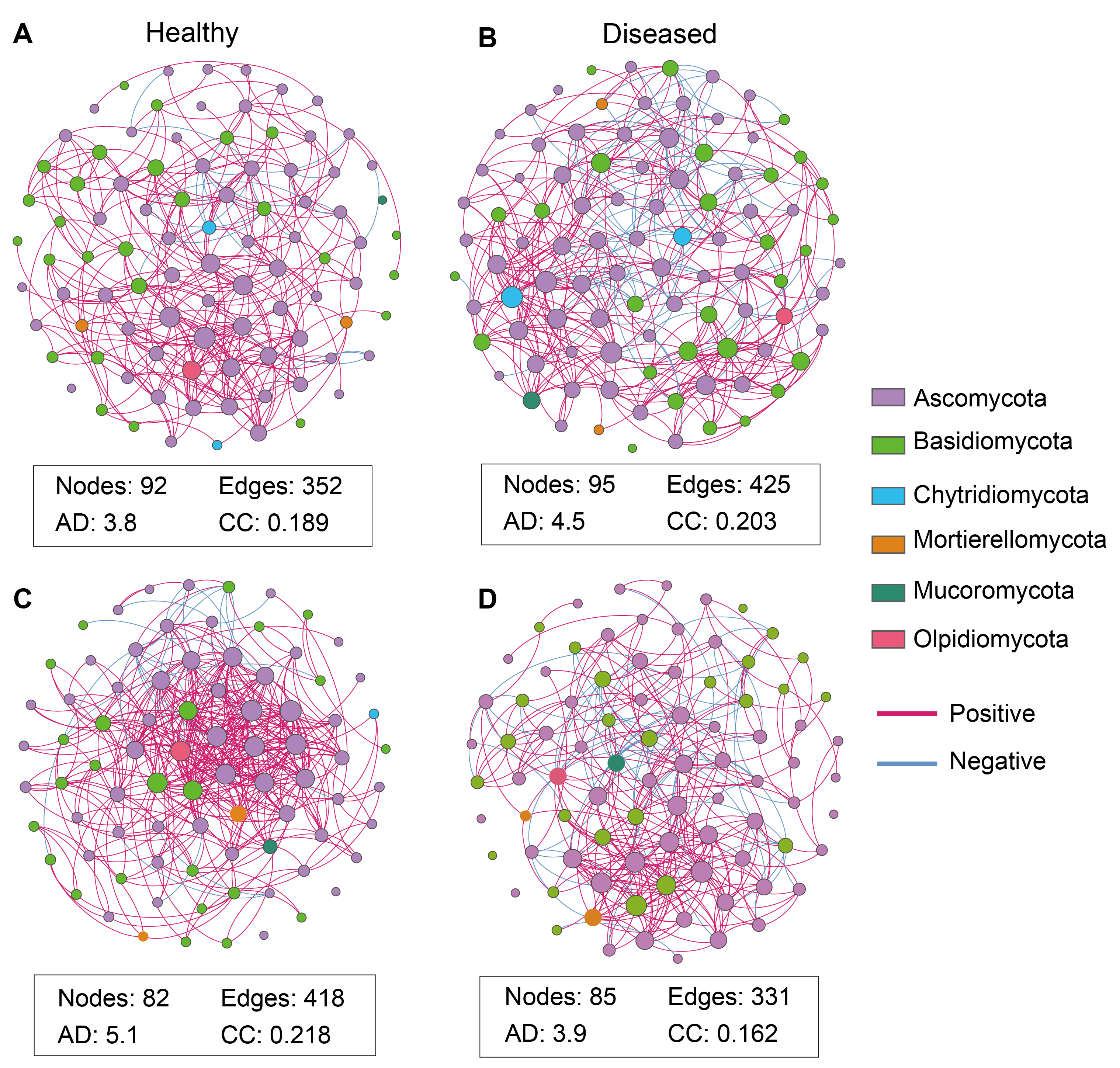

Supplement: Supplementary file 8 [file Image_7.tif]

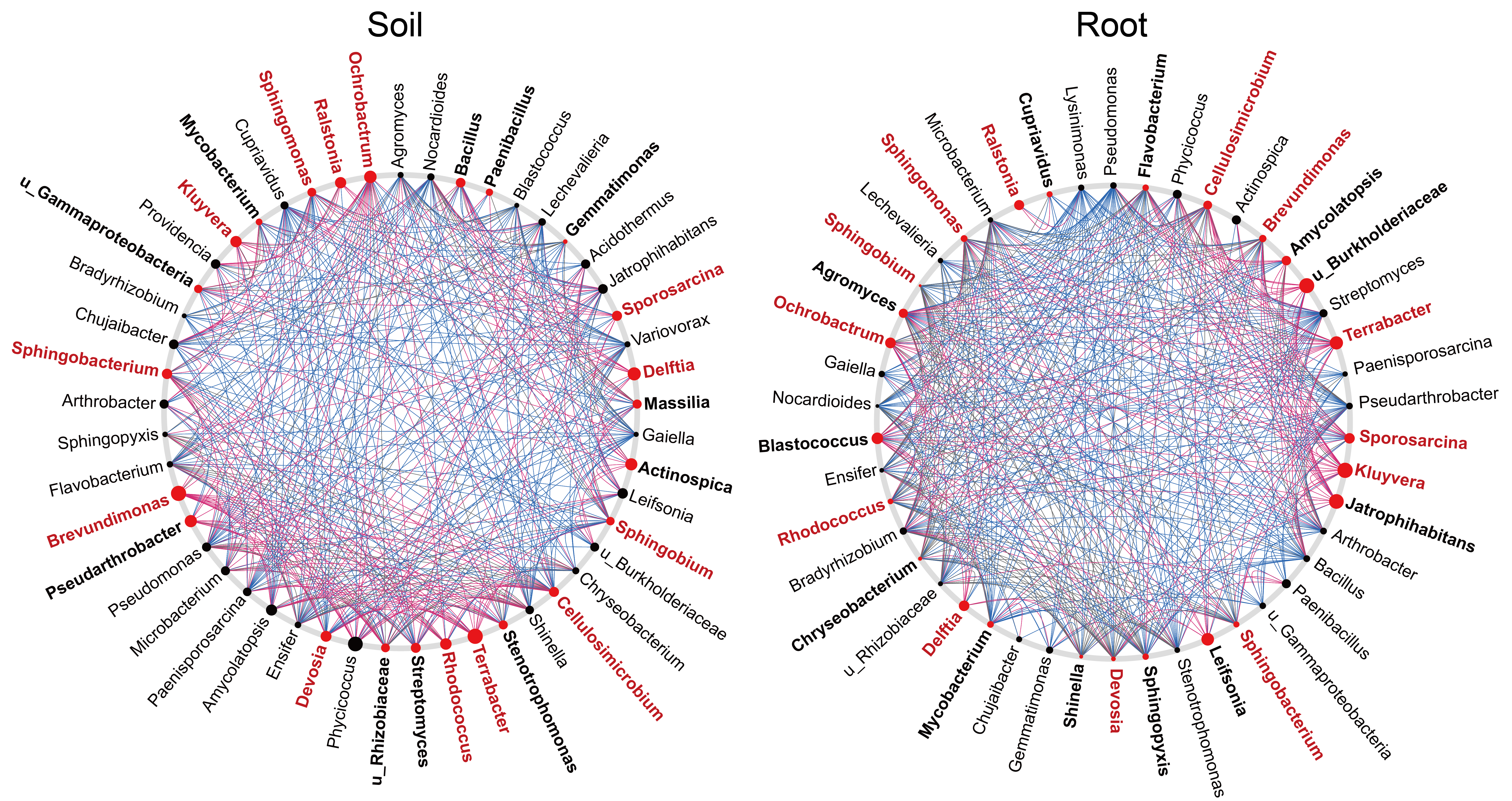

Supplement: Supplementary file 9 [file Image_8.tif]
